# Trigonal quasicrystalline states in $30^\circ$ rotated double moiré superlattices

**DOI:** 10.1038/s41598-021-91044-2

**Published:** 2021-06-02

**Authors:** J. A. Crosse, Pilkyung Moon

**Affiliations:** 1 New York University Shanghai, Arts and Sciences, Shanghai, 200122 China; 2 NYU-ECNU Institute of Physics at NYU Shanghai, Shanghai, 200062 China; 3 Department of Physics, New York University, New York, 10003 USA

**Keywords:** Electronic properties and materials, Surfaces, interfaces and thin films

## Abstract

We study the lattice configuration and electronic structure of a double moiré superlattice, which is composed of a graphene layer encapsulated by two other layers in a way such that the two hexagonal moiré patterns are arranged in a dodecagonal quasicrystalline configuration. We show that there are between 0 and 4 such configurations depending on the lattice mismatch between graphene and the encapsulating layer. We then reveal the resonant interaction, which is distinct from the conventional 2-, 3-, 4-wave mixing of moiré superlattices, that brings together and hybridizes twelve degenerate Bloch states of monolayer graphene. These states do not fully satisfy the dodecagonal quasicrystalline rotational symmetry due to the symmetry of the wave vectors involved. Instead, their wave functions exhibit trigonal quasicrystalline order, which lacks inversion symmetry, at the energies much closer to the charge neutrality point of graphene.

## Introduction

When two or more two-dimensional atomic layers which do not share a common periodicity are overlaid, an additional periodicity in the form of moiré interference pattern emerges^1^. The electronic structures of such systems—for example twisted bilayer graphene^2–6^, graphene on hexagonal boron nitride (hBN)^7–13^, and twisted bilayer transition metal dichalcogenides^14,15^ with small twist angles $$\theta \approx 1^\circ$$—have been investigated extensively. These materials have very long moiré superlattice vectors $$\mathbf{L}_i^{\mathrm{M}}$$ ($$i=1,2)$$, and, hence, exhibit many exotic properties such as the Fermi velocity renormalization^2,3^, mini Dirac points formation^11,13^, Hofstadter’s butterfly^5,7–9,16^, the emergence of superconductivity^17^, correlated phases^18^, and orbital magnetic moment^19^.

**Figure 1:**
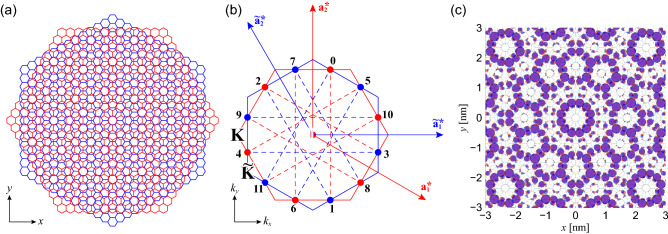
(**a**) Lattice structures of quasicrystalline twisted bilayer graphene^21,22,28^. The red and blue hexagons represent the unit cells of each layer. (**b**) The wave vectors of the twelve monolayer states $\mathbf{C}_n$ ($n=0,1,\ldots ,11$) which hybridize to quasicrystalline resonant states. The red and blue hexagons, and the red and blue arrows [$\mathbf{a}_i^*$ and $\tilde{\mathbf{a}}_i^*$ ($i=1,2$)] represent the first Brillouin zones and the reciprocal lattice vectors of each layer. Due to the symmetry, these twelve states are all degenerate in energy, and the dashed lines show that these twelve states interact by the reciprocal lattice vectors of the two layers. Note that these twelve states are centered around the $\Gamma$ point. (**c**) Local density of states of the quasicrystalline resonant states. The area of the circle is proportional to the squared wave amplitude, and red and blue circles represent the states in the upper and the lower layers, respectively.

A special case occurs when two hexagonal lattices are overlapped at $\theta =30^\circ$ (Fig. 1a). In this instance the atomic arrangement is mapped on to a quasicrystalline lattice, which is ordered but not periodic, with a 12-fold rotational symmetry^20–28^. Owing to the momentum mismatch^21^, quasicrystalline twisted bilayer graphene exhibits the electronic structure of almost decoupled bilayer graphene at most energy ranges. Nevertheless, it also hosts unique electronic states which satisfy the 12-fold rotational symmetry^22,27,28^. Such quasicrystalline states arise from the resonant interaction between the states at specific wave vectors via the rotational symmetry of the quasicrystal as well as the translational symmetry of the constituent atomic layers^22,28^. The red and blue hexagons in Fig. 1b show the first Brillouin zones of the two lattices. The numbered points and dashed lines show the wave vectors of the constituent monolayer states and the interlayer interaction which form the quasicrystalline resonant states. Such quasicrystalline states exhibit a wave amplitude distributed selectively on a limited number of sites in a characteristic 12-fold rotationally symmetric pattern (Fig. 1c). These states, however, appear at the energies (about $\pm 1.7\,\mathrm {eV})$ - far from the charge neutrality point of graphene. Similar quasicrystalline resonant states also arise in any bilayer stacked in a quasicrystalline configuration if all the dominant interlayer interactions occur between the atomic orbitals that have the same magnetic quantum number^28^. Thus, even transition metal dichalcogenides (TMDC) or square lattices can show the quasicrystalline states.

**Figure 2:**
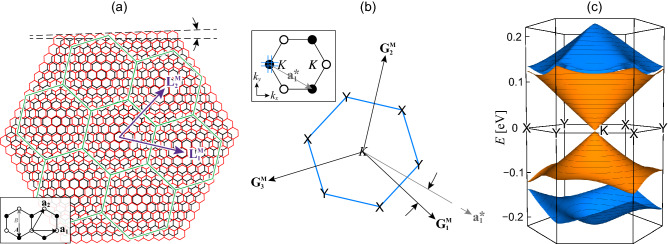
(**a**) Lattice structure of graphene on hBN^8,13^. The black and red hexagons represent the unit cells of graphene and hBN, respectively, and $\theta$ shows the relative orientation. The green hexagons and the vectors $\mathbf{L}_i^{\mathrm{M}}$ ($i=1,2$) represent the unit cells and superlattice vectors of the moiré superlattice, respectively. Here, the lattice constant of hBN is drawn 15% larger than that of graphene to enhance the visibility of the moiré pattern (actual difference is about 1.79%). Inset shows the lattice configuration of graphene; the black and white circles represent the *A* and *B* sublattices, $\mathbf{a}_i$ and $\varvec{\tau }_X$ show the primitive lattice vectors and the coordinates of the sublattices, respectively. (**b**) Superlattice Brillouin zone (blue hexagon) near the Dirac point (the region surrounded by blue lines in the inset) and the reciprocal lattice vectors $\mathbf{G}_i^{\mathrm{M}}$ of graphene on hBN. *X* and *Y* show the Brillouin zone corners where mini Dirac point appear, and $\phi$ shows the relative orientation of $\mathbf{G}_1^{\mathrm{M}}$ to the reciprocal lattice vector $\mathbf{a}_1^*$ of pristine graphene. Inset shows the first Brillouin zone of graphene, where the black and white circles represent the three equivalent Dirac points, *K* and $K'$, respectively. (**c**) The band dispersion of the first two bands in the conduction and valence bands of graphene on hBN with $\theta =0^\circ$, which show the band opening at the primary and the mini Dirac points.

Recently, rapid progress has been made in stacking more than two incommensurate atomic layers and a number of studies have investigated the effects of multiple moiré superlattice potentials on the electronic structure. The most notable example among them is a double moiré system, which is composed of a graphene layer encapsulated by hBN layers (BN/G/BN)^29,30^. The lattice mismatch between graphene and hBN results in a hexagonal moiré superlattice potential with a superlattice period $\mathbf{L}_i^{\mathrm{M}}$ ($i=1,2$) that can be as long as $14\,\mathrm {nm}$ (Fig. 2a). Such a long period [which results in short superlattice reciprocal lattice vectors, Fig. 2b] carves the graphene electronic structures into superlattice bands with an energy scale much smaller than that of pristine graphene (Fig. 2c)^13^. Recently, Leconte and Jung show that BN/G/BN at specific configurations can host two hexagonal moiré patterns overlaid at a twist angle of $30^\circ$, and claimed that the system hosts quasicrystalline electronic structure^31^. However, the interaction mechanism responsible for such unique electronic states in BN/G/BN, as well as the actual electronic band structure, and whether the wave functions actually satisfy the symmetry of the quasicrystal have not yet been investigated.

Here, we investigate the conditions where the two hexagonal *moiré patterns* in double moiré superlattice are arranged in a dodecagonal (12-fold) quasicrystalline configuration. Then we reveal the resonant interactions that bring together and hybridize twelve degenerate Bloch states of monolayer graphene and show that such interactions reconstruct the band dispersion of pristine graphene at these wave vectors. Compared to the resonant states of quasicrystalline twisted bilayer graphene where the actual *atomic lattices* are arranged in a dodecagonal configuration^22,27,28^, the resonant states of BN/G/BN appear at the energies much closer to the charge neutrality point of graphene. However, their wave functions show the quasicrystalline order with a three-fold rotational symmetry rather than fully satisfying the 12-fold rotational symmetry of the double moiré pattern.

## Methods

### Hexagonal moiré superlattices stacked at $30^\circ$

**Figure 3:**
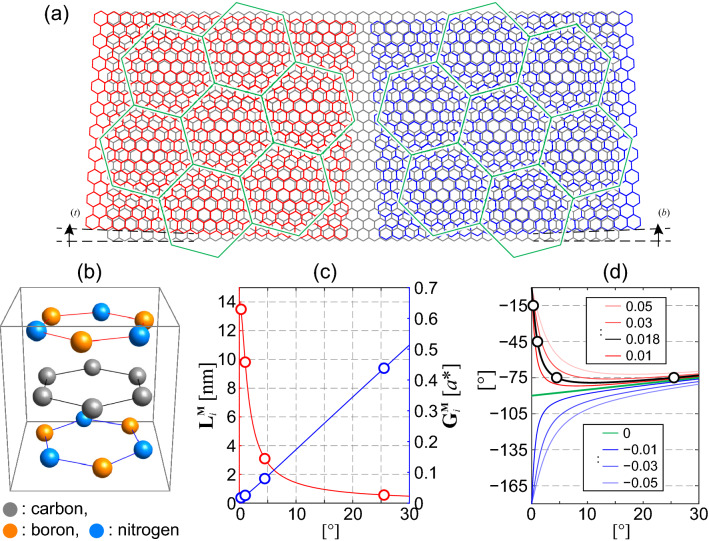
Lattice structure of graphene (gray hexagons) encapsulated by the top (red) and bottom (blue) hBN layers with twist angles of $\theta ^{(t)}$ and $\theta ^{(b)}$, respectively. Green hexagons show the unit cells of the moiré superlattices which are formed between the graphene and each of the hBN layer. We draw only the top (bottom) hBN layer at the left (right) side to enhance the visibility of the pattern. The two hexagonal superlattice unit cells are arranged at a relative angle of $30^\circ$, and form a dodecagonal quasicrystalline pattern when overlaid. (**b**) Atomic configuration of the three layers which shows the $D_3$ point group symmetry. (**c**) The lengths of the moiré lattice vectors (red line) and reciprocal lattice vectors (blue line) plotted against $\theta$. The circles correspond to the values at the configuration shown in Fig. 3(**a**). (**d**) The angle $\phi$ between the primitive vectors (both the real-space and the reciprocal lattice vectors) of graphene and moiré superlattice plotted against the twist angle $\theta$ between the two lattices. The red, green and blue lines show the plot for the systems with $\varepsilon >0$, $\varepsilon =0$ and $\varepsilon <0$, respectively. The black line corresponds to that between graphene and hBN ($\varepsilon =0.0179$). Black circles show the configuration that can form the quasicrystalline arrangement of the moiré patterns if the top and bottom hBN are rotated by $-\theta$ and $\theta$ from graphene, respectively.

We consider a trilayer system composed of graphene sandwiched by hBN. Both graphene and hBN are two-dimensional honeycomb lattice whose unit cell comprises of two (*A* and *B*) sublattices. Graphene has carbon atoms in both sublattices, while hBN has nitrogen atom on *A* site and boron atom on *B* site. The lattice constant of hBN, $\tilde{a} \approx 0.2504\,\mathrm {nm}$^32^, is slightly larger than that of graphene, $a \approx 0.246\,\mathrm {nm}$, and we use a constant interlayer distance of $d=0.322\,\mathrm {nm}$ between the adjacent two layers^33^. Here, we do not consider the lattice relaxation between graphene and hBN^34,35^, since the effects of such relaxation on the electronic structures is an order of a few meV. Nevertheless, our effective theory that respects the lattice symmetry is able to properly describe both the gap at the primary Dirac point and the asymmetric gap opening at the two inequivalent mini Dirac points^13^, as well as the orbital magnetism of the structure^19^.

We define the atomic structure of the double moiré superlattices by starting from a nonrotated arrangement, where the hexagon center of the three layers share the same in-plane position $(x,y)=(0,0)$, and the *A*-*B* bonds are parallel to each other. We choose $\mathbf{a}_1 = a(1,0)$ and $\mathbf{a}_2=a(1/2,\sqrt{3}/2)$ ($a=0.246\,\mathrm {nm}$) as the primitive lattice vectors of graphene, and $\varvec{\tau }_A=-\varvec{\tau }_1$ and $\varvec{\tau }_B=\varvec{\tau }_1$ [$\varvec{\tau }_1 = -(1/3)(\mathbf{a}_1-2\mathbf{a}_2)$] as the coordinates of the *A* and *B* sublattices in the unit cell. The primitive lattice vectors of the top ($l=t$) and the bottom ($l=b$) hBN layers become $\tilde{\mathbf{a}}_i^{(l)} = M \mathbf{a}_i$ ($i=1,2$), where $M= (1+\varepsilon ) \mathbb {I}$ represents the isotropic expansion by the factor $1+\varepsilon =\tilde{a}/a\approx 1.0179$, and $\varvec{\tau }_{\mathrm {N}}^{(l)}=-\varvec{\tau }_1^{(l)}\pm d \mathbf{e}_z$ and $\varvec{\tau }_{\mathrm {B}}^{(l)}=\varvec{\tau }_1^{(l)} \pm d\mathbf{e}_z$ [$\varvec{\tau }_1^{(l)} = -(1/3)(\tilde{\mathbf{a}}_1^{(l)}-2\tilde{\mathbf{a}}_2^{(l)})$], where the upper and lower signs are for the top and bottom layers, respectively, represent the coordinates of the nitrogen and boron atoms in the unit cell (Note that we defined the sublattice coordinates $\varvec{\tau }_X$ ($X=A,B$) and $\varvec{\tau }_{\tilde{X}^{(l)}}$ ($\tilde{X}=N,B$, $l=t,b$) differently from those in our previous work^13^, to make the points with the highest rotational symmetry, i.e., the hexagonal center, as the center of the system. However, both definitions keep the interaction matrices (Eq. 9) the same.). We define the reciprocal lattice vectors $\mathbf{a}_i^*$ and $\tilde{\mathbf{a}}_i^*$ for graphene and hBN, respectively, so as to satisfy $\mathbf{a}_i \cdot \mathbf{a}_j^* = \tilde{\mathbf{a}}_i \cdot \tilde{\mathbf{a}}_j^* = 2\pi \delta _{ij}$. We then rotate the top and bottom hBN layers with respect to graphene by arbitrary angles $\theta ^{(t)}$ and $\theta ^{(b)}$ around the origin, respectively. From now on, we use “BN/G/BN” for this configuration only. Due to the symmetry of the lattice, $0\le \theta ^{(l)} \le 30^\circ$ ($l\in t,b$) spans all the independent configurations.

Figure 3a shows the moiré interference patterns which arise from the lattice mismatch between the top hBN and graphene (left side), and also that from the bottom hBN and graphene (right side), respectively, and Fig. 3b shows the atomic configuration of the three layers. The lattice vectors $\mathbf{L}_i^{\mathrm {M},(l)}$ and the reciprocal lattice period $\mathbf{G}_i^{\mathrm {M},(l)}$ ($i=1,2$) of each moiré superlattice are

1 $$\begin{aligned} \mathbf{L}_i^{\mathrm {M},(l)}&=c R(\phi ^{(l)}) \mathbf{a}_i, \nonumber \\ \mathbf{G}_i^{\mathrm {M},(l)}&=c^{-1} R(\phi ^{(l)}) \mathbf{a}^*_i, \end{aligned}$$

respectively, where $c=(1+\varepsilon )/\sqrt{\varepsilon ^2+2(1+\varepsilon )(1-\cos \theta ^{(l)})}$, $\phi ^{(l)} = \mathrm {arctan} [-\sin \theta ^{(l)}/(1+\varepsilon -\cos \theta ^{(l)})]$, and $R(\phi )$ is a rotation by $\phi$^13,36^. We plot $|\mathbf{L}_i^{\mathrm {M}}|$ and $|\mathbf{G}_i^{\mathrm {M}}|$ against $\theta$ in Fig. 3c in red and blue lines, respectively.

**Figure 4:**
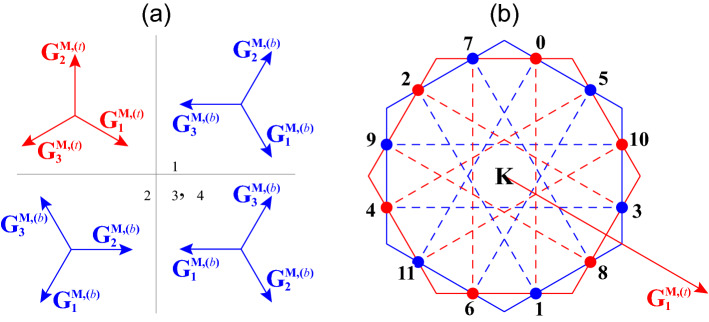
(**a**) Relative orientation of the moiré reciprocal lattice vectors of the lower moiré superlattice ($\mathbf{G}_i^{\mathrm {M},(b)}$, blue arrows) for $\theta _i$ ($i=1,2,3,4$) with respect to the direction of the vectors of the upper moiré superlattice ($\mathbf{G}_i^{\mathrm {M},(t)}$, red arrows). (**b**) Relative direction of the wave vectors involved in the resonant coupling [showing the number *n* of $\mathbf{C}_n$, Eq. (14)] with respect to the direction of $\mathbf{G}_i^{\mathrm {M},(t)}$. In both figures, note that the actual direction of $\mathbf{G}_1^{\mathrm {M},(t)}$ is $\phi$ rotated from $\mathbf{a}_1^*$ (Eq. 1).

Now, we will find the configuration where the unit cells of the two hexagonal moiré superlattices have the same size and are overlaid with a relative twist angle of $30^\circ$. In such a configuration, the overlaid two hexagonal superlattices are mapped onto a 12-fold rotationally symmetric quasicrystalline lattice without any translational symmetry, as first shown by Stampfli^37^. From Eq. (1), the former and the latter conditions give $|\theta ^{(t)}|=|\theta ^{(b)}|$ and $\phi ^{(t)}-\phi ^{(b)} \equiv 30^\circ \,(\mathrm {mod}\,60^\circ )$, which can be simultaneously satisfied by $\theta ^{(t)}=-\theta ^{(b)}$ and $\phi ^{(t)}=-\phi ^{(b)}\equiv 15^\circ \,(\mathrm {mod}\,30^\circ )$. Figure 3d shows $\phi$ as a function of $\theta$ for various $\varepsilon$. The red, green, blue lines correspond to $\varepsilon >0$, $\varepsilon =0$, $\varepsilon <0$, respectively, and the thick black line corresponds to hBN. The two hexagonal moiré superlattices form a dodecagonal quasicrystalline configuration at $\theta$ where the line and the dashed horizontal lines cross. If the lattice constant of the top and bottom layers is the same as that of the middle, graphene layer, i.e., $\varepsilon =0$, then the two hexagonal moiré patterns cannot have a relative twist angle of $30^\circ$. On the other hands, the systems with $\varepsilon <0$, $0<\varepsilon < 0.0353$, and $\varepsilon \ge 0.0353$ have three, four, and two $\theta$ which satisfy the conditions. When the top and bottom layers are hBN, i.e., $\varepsilon \approx 0.0179$, the four angles are $\theta _1=0.274^\circ$, $\theta _2=1.03^\circ$, $\theta _3=4.48^\circ$, $\theta _4=25.5^\circ$, and the corresponding $|\mathbf{G}_i^{\mathrm{M}}|$ are 0.0182, 0.0251, 0.0795, 0.438 times the $|\mathbf{a}_i^*|$. Note that $\theta _4$ gives very long $|\mathbf{G}_i^{\mathrm{M}}|$, and accordingly very short $|\mathbf{L}_i^{\mathrm{M}}|$, which competes with the length scale of monolayer graphene. By choosing $\theta ^{(t)}=-\theta _i$ and $\theta ^{(b)}=\theta _i$ ($i=1,2,3,4$), we get $\phi ^{(t)}=-\phi _i$ and $\phi ^{(b)}=\phi _i$, where $\phi _1=-15^\circ$, $\phi _2=-45^\circ$, and $\phi _3=\phi _4=-75^\circ$. Then, the twelve moiré reciprocal lattice vectors

2 $$\begin{aligned} \{\pm \mathbf{G}_i^{\mathrm {M},(l)} \,| \,i=1,2,3, \,l=t,b\}, \end{aligned}$$

where $\mathbf{G}_3^{\mathrm {M},(l)} = -\mathbf{G}_1^{\mathrm {M},(l)}-\mathbf{G}_2^{\mathrm {M},(l)}$, are arranged in 12-fold rotational symmetry (Fig. 4a), just like the reciprocal lattice vectors in quasicrystalline twisted bilayer graphene that give rise to the resonant states (Fig. 1b)^22^.

It should be noted that, however, although the overlap of the two moiré interference patterns are mapped onto a quasicrystalline tiling with 12-fold rotational symmetry, the actual lattice structure belongs to the symmetry group $D_3$; it is invariant under $C_3$ rotation about the axis perpendicular to the *xy*-plane and under three $C_2$ rotation about the axes in the plane, but lacks inversion symmetry. If we replace the top and bottom hBN layers by a material having the same types of atoms in both sublattices, then the lattice has the symmetry group $D_6$ which is still lower than the 12-fold rotational symmetry.

### Hamiltonian of double moiré superlattices

The total tight-binding Hamiltonian of the double moiré superlattice is expressed as

3 $$\begin{aligned} H = H_{\mathrm{G}} + H_{\mathrm {hBN}}^{(t)} + H_{\mathrm{hBN}}^{(b)} + U^{(t)} + U^{(l)}, \end{aligned}$$

where $H_{\mathrm{G}}$ and $H_{\mathrm{hBN}}^{(t)}$ ($H_{\mathrm{hBN}}^{(b)}$) represent the Hamiltonian for the intrinsic monolayer graphene and the top (bottom) hBN, respectively, $U^{(t)}$ ($U^{(b)}$) is for the interlayer coupling between the graphene and the top (bottom) hBN. However, since the hBN electronic bands are far from the charge neutrality point of graphene, we can project the total Hamiltonian on to the Bloch bases of graphene $p_z$ orbitals at each sublattice,

4 $$\begin{aligned} |\mathbf{k},X\rangle = \frac{1}{\sqrt{N}}\sum _{\mathbf{R}_{X}} e^{i\mathbf{k}\cdot \mathbf{R}_{X}} |\mathbf{R}_{X} \rangle \end{aligned}$$

where $|\mathbf{R}_{X} \rangle$ is the atomic orbital at the site $\mathbf{R}_{X}=n_1 \mathbf{a}_1 + n_2 \mathbf{a}_2 + \varvec{\tau }_X$ ($n_i \in \mathbb {Z}$, $X=A,B$), $\mathbf{k}$ is the two-dimensional Bloch wave vectors and $N = S_{\mathrm{tot}}/S$ is the number of the graphene unit cells with an area $S=(\sqrt{3}/2)a^2$ in the total system area $S_{\mathrm{tot}}$. Then, the Hamiltonian near the Dirac point $K^\xi = -\xi (2\mathbf{a}_1^*+\mathbf{a}_2^*)/3$ of graphene, where $\xi =\pm 1$ for *K* and $K'$, respectively, is reduced to a $2\times 2$ form^13^ (Note that the theoretical model can be easily expanded to the systems with the encapsulating layers other than hBN; e.g., it is straightforward to expand the model to the atomic layers of which energy bands are close to the charge neutrality point of graphene by explicitly using the Bloch bases for those layers.),

5 $$\begin{aligned} \tilde{H}&= H_{\mathrm{G}} + U^{(t)\dagger } (-H_{\mathrm{hBN}}^{(t)})^{-1} U^{(t)}+ U^{(b)\dagger } (-H_{\mathrm{hBN}}^{(b)})^{-1} U^{(b)} \nonumber \\&\equiv H_{G} + V_{\mathrm{hBN}}^{(t)}+ V_{\mathrm{hBN}}^{(b)}. \end{aligned}$$

The intralayer matrix elements of graphene are given by

6 $$\begin{aligned} H_{\mathrm{G}}&= \begin{pmatrix} h_{AA} &{} h_{AB} \\ h_{BA} &{} h_{BB} \end{pmatrix}, \nonumber \\ h_{X,X'}(\mathbf{k})&= \sum _{\mathbf{L}} -T(\mathbf{L}+\varvec{\tau }_{X'X}) e^{-i\mathbf{k}\cdot (\mathbf{L}+\varvec{\tau }_{X'X})}, \end{aligned}$$

where $$\mathbf{L} = n_1 \mathbf{a}_1 + n_2 \mathbf{a}_2$$, $$\varvec{\tau }_{X'X} = \varvec{\tau }_{X'}- \varvec{\tau }_{X}$$, and

7 $$\begin{aligned} -T(\mathbf{R})&= V_{pp\pi }\left[ 1-\left( \frac{\mathbf{R}\cdot \mathbf{e}_z}{R}\right) ^2\right] + V_{pp\sigma }\left( \frac{\mathbf{R}\cdot \mathbf{e}_z}{R}\right) ^2, \nonumber \\ V_{pp\pi }&= V_{pp\pi }^0 e^{- (R-a/\sqrt{3})/r_0}, \quad V_{pp\sigma } = V_{pp\sigma }^0 e^{- (R-d)/r_0}, \end{aligned}$$

is the transfer integral between two $p_z$ orbitals at a relative vector $\mathbf{R}$^5,38^, $V_{pp\pi }^0 \approx -3.38\,\mathrm {eV}$ (Note that the value of $V_{pp\pi }^0 \approx -3.38~\mathrm{eV}$ used in this work is different from that used in the previous works ($V_{pp\pi }^0 \approx -2.7~\mathrm{eV}$) on the twisted bilayer graphene^6,13,39^ and graphene on hexagonal boron nitride^13^. In this work, we scaled $V_{pp\pi }^0$ by a factor of 1.25 to compensate the deviation of the Fermi velocity of a pristine graphene, which affects the entire energy scale, due to the summation over sites in the hopping range.), $V_{pp\sigma }^0 \approx 0.48\,\mathrm {eV}$, and $r_0 \approx 0.0453\,\mathrm {nm}$^6,39^. The effective potentials by hBN to graphene, $V_{\mathrm{hBN}}^{(l)}$, are explicitly written as^13^ (Note that we defined the sublattice coordinates $\varvec{\tau }_X$ ($X=A,B$) and $\varvec{\tau }_{\tilde{X}^{(l)}}$ ($\tilde{X}=N,B$, $l=t,b$) differently from those in our previous work^13^, to make the points with the highest rotational symmetry, i.e., the hexagonal center, as the center of the system. However, both definitions keep the interaction matrices (Eq. 9) the same.)

8 $$\begin{aligned} V_{\mathrm{hBN}}^{(l)} = W_0 + \{ W_1^\xi e^{i\xi \mathbf{G}_1^{\mathrm {M},(l)} \cdot \mathbf{r}}+ W_2^\xi e^{i\xi \mathbf{G}_2^{\mathrm {M},(l)} \cdot \mathbf{r}}+ W_3^\xi e^{i\xi \mathbf{G}_3^{\mathrm {M},(l)} \cdot \mathbf{r}}+\mathrm {h.c.}\}, \end{aligned}$$

where we truncated much weaker terms $\mathcal {O}(u_0^4)$ which are associated with longer momentum difference. Here,

9 $$\begin{aligned} W_0 = V_0 \begin{pmatrix} 1 &{} 0 \\ 0 &{} 1 \end{pmatrix},\quad W_1^\xi = V_1 e^{i\xi \psi } \begin{pmatrix} 1 &{} \omega ^{-\xi } \\ 1 &{} \omega ^{-\xi } \end{pmatrix},\quad W_2^\xi = V_1 e^{i\xi \psi } \begin{pmatrix} 1 &{} \omega ^{\xi } \\ \omega ^{\xi } &{} \omega ^{-\xi } \end{pmatrix},\quad W_3^\xi = V_1 e^{i\xi \psi } \begin{pmatrix} 1 &{} 1 \\ \omega ^{-\xi } &{} \omega ^{-\xi } \end{pmatrix}, \end{aligned}$$

and

10 $$\begin{aligned} V_0 &= -3 u_0^2 \left( 
\frac{1}{V_{\mathrm{N}}} + \frac{1}{V_{\mathrm{B}}} \right) , \nonumber \\ V_1 e^{i\xi \psi }&= - u_0^2 \left( \frac{1}{V_{\mathrm{N}}} + \omega ^\xi \frac{1}{V_{\mathrm{B}}} \right) , \end{aligned}$$

where $\omega = e^{2\pi i/3}$, and $u_0 \approx -t(K^\xi ) \approx 0.152\,\mathrm {eV}$ is the in-plane Fourier transformation of the transfer integral between two $p_z$ orbitals (Eq. 7)

11 $$\begin{aligned} t(\mathbf{q}) = \frac{1}{S} \int T(\mathbf{r}+ z_{\tilde{X}X}\mathbf{e}_z) e^{-i \mathbf{q}\cdot \mathbf{r}} d\mathbf{r} \end{aligned}$$

at $\mathbf{q}$ near the Dirac point^5,38^. We will discuss more about $u_0$ later. By using $V_{\mathrm {C}}=0$, $V_{\mathrm {N}}=-1.40\,\mathrm {eV}$, and $V_{\mathrm {B}}=3.34\,\mathrm {eV}$, as the on-site potential of carbon, nitrogen, and boron atoms, respectively^40^, we get $V_0 \approx 0.0289\,\mathrm {eV}$, $V_1 \approx 0.0210\,\mathrm {eV}$, and $\psi \approx -0.29$ (rad). If we replace the top and bottom hBN layers by a material having the same types of atoms in both sublattices, i.e., if $V_{\mathrm {N}}=V_{\mathrm {B}}$, then $\psi \equiv \pi /3 \,(\mathrm {mod}\,\pi )$. The symmetry of such a structure increases to $D_6$, and the reduced Hamiltonian $\tilde{H}$ gains the inversion symmetry

12 $$\begin{aligned} \tilde{H}^{(-\xi )}(\mathbf{k},\mathbf{r})=\sigma _x [\tilde{H}^{(\xi )}(-\mathbf{k},-\mathbf{r})] \sigma _x. \end{aligned}$$

It is straightforward to show that the reduced Hamiltonian $\tilde{H}$ spans the subspace

13 $$\begin{aligned} \{|\mathbf{k},X\rangle \,| \, \mathbf{k}=\mathbf{k}_0+\sum \limits _{l=t,b} \sum \limits _{i=1,2,3} m_i^{(l)} \mathbf{G}_i^{\mathrm {M},(l)}, \, m_i^{(l)}\in \mathbb {Z} \}, \end{aligned}$$

for any $\mathbf{k}_0$ in the momentum space. To investigate the electronic structures near $\mathbf{k}_0$, for any practical calculation, we only need a limited number of bases around $\mathbf{k}_0$ inside a certain cut-off circle $k_c$, because the interaction with the states far from $\mathbf{k}_0$ is very weak due to multiple scattering. We can, then, obtain the energy eigenvalues at all the wave vectors in Eq. (13) by diagonalizing the Hamiltonian matrix within the finite bases, and the quasiband dispersion of the system by plotting the energy levels against $\mathbf{k}_0$. Here the wave number $\mathbf{k}_0$ works like the crystal momentum for the periodic system, and so it can be called the quasicrystal momentum.

### Quasicrystalline resonant interaction by two moiré superlattice potentials

In addition to the typical 2- and 3-wave interaction by each moiré superlattice (Fig. 2c)^13^, the 12-fold rotational symmetry of the wave vectors which couple the monolayer states (Eq. 2) as well as the translational symmetries of the two moiré superlattices enables a unique interaction between twelve degenerate monolayer states. Such a resonant coupling occurs at the twelve symmetric points

14 $$\begin{aligned} \mathbf{C}_n =K^\xi + 2|\mathbf{G}_i^{\mathrm {M},(l)}| \sin (\pi /12) (\cos \theta _n, \sin \theta _n), \end{aligned}$$

shown in Fig. 4b, where $\theta _n = \frac{5\pi }{12} + \frac{7n\pi }{6} - \phi _i$ ($n=0,1,2,\cdots ,11$). While the twelve waves which constitute the resonant states in quasicrystalline twisted bilayer graphene are centered around the $\Gamma$ point^28^, the waves involved in the resonant interaction in BN/G/BN are centered around the Dirac point $K^\xi$. These twelve states are degenerate if we ignore the small trigonal warping in this low energy regime. We see that the states at $\mathbf{C}_i$ strongly interact with the states at $\mathbf{C}_{i-1}$ and $\mathbf{C}_{i+1}$ by the reciprocal lattice vectors of the top and bottom moiré superlattices. The interaction with states at any other $\mathbf{k}$ can be safely neglected since the interaction strength is much less or the two states are not degenerate in most cases. Hence, these states form one-dimensional monatomic chain with twelve sites and two pseudospins whose interaction is described by the moiré potential (Eq. 5).

It should be noted that this is not the only resonant coupling in this system. As shown in previous work on quasicrystalline twisted bilayer graphene (e.g., Appendix A in Ref.^28^), there are more sets of states, with different wave numbers, that show the resonant interaction between the constituent monolayer states. However, the set in Fig. 4b is associated with the strongest interaction $|t(\mathbf{q})|$ and, hence, gives the largest energy separation between the hybridized states.

## Results and discussion

By using the Bloch bases ($|\mathbf{k}^{(0)}\rangle$, $|\mathbf{k}^{(1)}\rangle$, $\,\cdots$, $|\mathbf{k}^{(11)}\rangle$) near the twelve wave vectors $\mathbf{k}^{(n)}=\mathbf{C}_n+\mathbf{k}_0$, where $|\mathbf{k}^{(n)}\rangle$ is $(|\mathbf{k}^{(n)},A\rangle ,\eta |\mathbf{k}^{(n)},B\rangle )$ with $\eta =\omega ^{\xi \times \mathrm {floor}(n/4)}$, we can express the Hamiltonian of the resonant ring $H_{\mathrm {ring}}^\xi$ by a $24\times 24$ matrix

15 $$\begin{aligned} H_{\mathrm{ring}}^\xi (\mathbf{k}_0) = \left( \begin{array}{cccccccccccc} H_0^{(0)} &{} W_2^\xi &{}&{}&{}&{}&{}&{}&{}&{}&{}&{} Y_1^{\xi \dagger } \\ W_2^{\xi \dagger } &{} H_1^{(0)} &{} X_2^{\xi \dagger } \\ &{} X_2^\xi &{} H_2^{(0)} &{} W_1^{\xi \dagger } \\ &{}&{} W_1^\xi &{} H_3^{(0)} &{} Y_1^\xi \\ &{}&{}&{} Y_1^{\xi \dagger } &{} H_0^{(4)} &{} W_2^\xi \\ &{}&{}&{}&{} W_2^{\xi \dagger } &{} H_1^{(4)} &{} X_2^{\xi \dagger } \\ &{}&{}&{}&{}&{} X_2^{\xi } &{} H_2^{(4)} &{} W_1^{\xi \dagger } \\ &{}&{}&{}&{}&{}&{} W_1^{\xi } &{} H_3^{(4)} &{} Y_1^{\xi } \\ &{}&{}&{}&{}&{}&{}&{} Y_1^{\xi \dagger } &{} H_0^{(8)} &{} W_2^{\xi } \\ &{}&{}&{}&{}&{}&{}&{}&{} W_2^{\xi \dagger } &{} H_1^{(8)} &{} X_2^{\xi \dagger } \\ &{}&{}&{}&{}&{}&{}&{}&{}&{} X_2^{\xi } &{} H_2^{(8)} &{} W_1^{\xi \dagger } \\ Y_1^\dagger &{}&{}&{}&{}&{}&{}&{}&{}&{}&{} W_1^{\xi } &{} H_3^{(8)} \\ \end{array}\right) . \end{aligned}$$

Here

16 $$\begin{aligned} H_i^{(n)}&= \begin{pmatrix} h_{AA}^{(n,i)} &{} h_{AB}^{(n,i)} \\ h_{BA}^{(n,i)} &{} h_{BB}^{(n,i)} \\ \end{pmatrix} + 2W_0, \nonumber \\ h_{X'X}^{(n,i)}(\mathbf{k}_0)&= h_{X'X}[R(-7n\pi /6) \mathbf{k}_0 + \mathbf{C}_i], \end{aligned}$$

and

17 $$\begin{aligned} X_2^\xi = \left\{ \begin{array}{l} W_1^{\xi \dagger }\qquad \mathrm {for\;\theta _{1}} \\ W_3^\xi \qquad \mathrm {}for\;\theta _{2} \\ W_2^{\xi \dagger } \qquad \mathrm {for}\;\theta _{3}\;\mathrm {and}\;\theta _{4} \end{array}\right. \qquad \qquad Y_1^\xi = \left\{ \begin{array}{l} V_1 e^{-i\xi \psi } \begin{pmatrix} 1 &{} \omega ^{-\xi } \\ 1 &{} \omega ^{-\xi } \end{pmatrix}\qquad \mathrm {for}\;\theta _{1} \\ V_1 e^{i\xi \psi } \begin{pmatrix} 1 &{} \omega ^{-\xi } \\ \omega ^{\xi } &{} 1 \end{pmatrix}\qquad \mathrm {for}\;\theta _{2} \\ V_1 e^{-i\xi \psi } \begin{pmatrix} 1 &{} \omega ^{\xi } \\ \omega ^\xi &{} \omega ^{-\xi } \end{pmatrix} \qquad \mathrm {for}\;\theta _{3}\;\mathrm {and}\;\theta _{4} \end{array}\right. \end{aligned}$$

for $\theta _1$, $\theta _2$, and both $\theta _3$ and $\theta _4$, respectively.

Obviously, Eq. (15) is symmetric under rotation by *four* span of the ring (i.e., moving $\mathbf{C}_n$ to $\mathbf{C}_{n+4}$), which actually corresponds to the $[R(7\pi /6)]^4$ ($120^\circ$ rotation) of the entire system. This means that the resonant states of the BN/G/BN have a three-fold rotational symmetry, which is much lower than the 12-fold rotational symmetry of the double moiré patterns of the system. This Hamiltonian cannot obtain a six-fold rotational symmetry, even if we replace the top and bottom hBN layers by a material having the same types of atoms in both sublattices (e.g., $V_{\mathrm {N}}=V_{\mathrm {B}}$), since (i) atomic structure lacks 12-fold rotational symmetry and (ii) the 12 wave vectors involved are centered at $K^\xi$ which has three-fold rotational symmetry (Fig. 2b). Thus, the relevant terms cannot be gauged out by a similarity transformation. There is, however, an exception in the systems with $V_{\mathrm {N}}=V_{\mathrm {B}}$; the wave functions at $\mathbf{k} _0=\mathbf{0}$, and only at this $\mathbf{k} _0$, show a six-fold rotational symmetry (Fig. 6d–f).

The interlayer interaction strength $u_0$ (Eq. 10) deviates from $-t(K^\xi )$ as the distance between $\mathbf{k}^{(n)}$ and $K^\xi$ increases. This effect becomes obvious in the system with a longer $|\mathbf{G}_i^{\mathrm {M},(l)}|$, e.g., in BN/G/BN with $\theta _4$. However, what is more important is that, the $u_0$ associated with the interaction between the neighboring Bloch states $|\mathbf{k}^{(n)}\rangle$ ($n=0,1,\cdots ,11$) [dashed lines in Fig. 4b] are not the same. Since the Fourier transformation of the transfer integral between $p_z$ orbitals, $t(\mathbf{q})$ (Eq. 11), are isotropic along the in-plane direction, $t(\mathbf{q})$ depends only on $|\mathbf{q}|$. If any electronic state is mainly comprised of three monolayer states of which waves vectors are arranged in a three-fold rotationally symmetric way around $K^\xi$, e.g., $K^\xi +\mathbf{k}$, $K^\xi +R(2\pi /3)\mathbf{k}$, $K^\xi +R(4\pi /3)\mathbf{k}$, then the $|\mathbf{q}|$ associated with the interaction between the monolayer states are identical since we can freely choose one among the three equivalent $K^\xi$, i.e., $K^\xi$, $R(2\pi /3)K^\xi$, $R(4\pi /3)K^\xi$, for each state. This is the case that happens at the mini Dirac point (the hexagonal Brillouin zone corners) of graphene on hBN^13^. On the contrary, $t(\mathbf{q})$ for each interaction of the resonant coupling in BN/G/BN are not identical, since there are more than three states involved. As a result, $u_0^2$ in Eq. (10) varies $\pm 2,3\%$ for $\theta _1$, $\theta _2$, $\theta _3$, and $\pm 11\%$ for $\theta _4$. Nevertheless, since the twelve wave vectors are arranged in a 12-fold rotationally symmetric way, equally spaced triplets (i.e., $n\in \{0, 4, 8\}$, $n\in \{1, 5, 9\}$, $n\in \{2,6,10\}$, $n\in \{3,7,11\}$) satisfy the three-fold rotational symmetry with $K^\xi$. As a result, the oscillation of $u_0^2$ is consistent with the rotational symmetry of $H_{\mathrm {ring}}^\xi$ and does not reduce the three-fold symmetry further. Hereafter, we will consider the structures with $\theta _1$, $\theta _2$, $\theta _3$ only, and use $u_0=-t(K^\xi )\approx 0.152\,\mathrm {eV}$, i.e., ignore the variation of $u_0$.

Since a hBN layer lacks the inversion symmetry, it is natural to ask whether the band structure changes if we rotate one of the hBN layers by $180^\circ$ (We label this structure BN/G/NB). The BN/G/NB also belongs to the symmetry group $D_3$, while the three $C_2$ axes are $60^\circ$ rotated from those of BN/G/BN. It is well known that twisted double bilayer graphene^41–44^ and BN/G/BN at general angles^31^ show the change of electronic structures with respect to such a change. The $180^\circ$ rotation of hBN corresponds to the swap of the boron and nitrogen atoms. Thus, we can get the effective potential Eq. (8) of the moiré superlattice from such a layer by replacing $\psi$ by $-\psi +2\pi /3$ (Eq. 10), while keeping that of the other moiré superlattice unchanged. However, we can reduce the Hamiltonian of BN/G/NB to that of BN/G/BN (Eq. 15) by a similarity transformation that multiplies $e^{2i\xi \psi }\omega ^{-\xi }$ ($e^{-2i\xi \psi }\omega ^{\xi }$) to the Bloch bases $|\mathbf{k}^{(n)}\rangle$ with $n\equiv 2,3 \,(\mathrm {mod}\,4)$ for $\theta _1$, $\theta _3$, $\theta _4$ ($\theta _2$). As a result, the resonant states are invariant with respect to the replacement of BN to NB.

**Figure 5:**
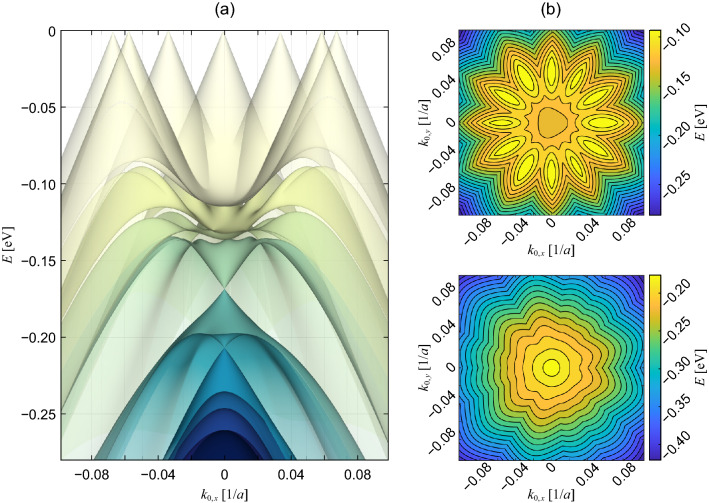
(**a**) Electronic structure in the valence band side of the $30^\circ$ rotated double moiré superlattice at $\theta _1$. (**b**) Energy contours of the third (top panel) and the sixth (bottom panel) valence bands which clearly show the three-fold rotational symmetry.

Figure 5a shows the valence band structures of BN/G/BN at $\theta _1$ near the resonant states at $\mathbf{C}_n$ plotted as a function of $\mathbf{k}_0$. The twelve Dirac cones are arranged on a circle with a radius $\Delta k = 2|\mathbf{G}_i^{\mathrm {M},(l)}| \sin (\pi /12)$ and they are strongly hybridized near $\mathbf{k}_0=\mathbf{0}$ to exhibit the characteristic dispersion including parabolic bottoms, a frilled band edge, and new Dirac points at $-0.164\,\mathrm {eV}$ and $-0.203\,\mathrm {eV}$. We have similar resonant states also in the conduction band, while the energy spacing between the resonant states is much smaller than in the valence band, just like the cases of graphene on hBN^13^ and quasicrystalline twisted bilayer graphene^22^. As predicted by the symmetry of $H_{\mathrm {ring}}^\xi$, the band structures exhibit three-fold rotational symmetry around $\mathbf{k}_0=\mathbf{0}$, as we can clearly see from the energy contours in Fig. 5b. The structures with the other angles, $\theta _i$ ($i=2,3,4$), also show similar band dispersion, except that the resonant states are formed at the energies far from the charge neutrality point of graphene, since they have longer $|K^\xi -\mathbf{C}_n|$. The energy splitting between the resonant states in BN/G/BN, $\mathcal {O}(|V_1|)$, is much smaller than that in the quasicrystalline twisted bilayer graphene, $\mathcal {O}(|u_0|)$, since the interaction here involves a second order scattering through the hBN layer. Nevertheless, the resonant states of BN/G/BN appear at the energies much closer to the charge neutrality point of graphene than those of quasicrystalline twisted bilayer graphene (about $\pm 1.7\,\mathrm {eV})$, since the wave vectors responsible for the interaction in BN/G/BN ($\mathcal {O}(|\mathbf{G}_i^{\mathrm {M},(l)}|)$) are much shorter than those in quasicrystalline twisted bilayer graphene ($\mathcal {O}(|\mathbf{a}_i^*|)$). Thus, the resonant states of BN/G/BN appear at much smaller, experimentally feasible, electron densities.

At $\mathbf{k}_0=\mathbf{0}$, we can reduce $H_{\mathrm {ring}}^\xi$ to an $8\times 8$ form,

18 $$\begin{aligned} H_{\mathrm{ring}}^{(\xi ,m)}(\mathbf{k}_0) = \begin{pmatrix} H_0 &{} W_2^\xi &{} 0 &{} Y_1^{\xi \dagger } \omega ^{-m} \\ W_2^{\xi \dagger } &{} H_1 &{} X_2^{\xi \dagger } &{} 0\\ 0 &{} X_2^\xi &{} H_2 &{} W_1^{\xi \dagger } \\ Y_1^\xi \omega ^m &{} 0&{} W_1^\xi &{} H_3 \\ \end{pmatrix}, \end{aligned}$$

by using the Bloch condition along the one-dimensional chain. Here, $H_i=H_i^{(0)}(\mathbf{k}_0=\mathbf{0})$ ($i=0,1,2,3$) and $m=-1,0,1$ is the quantized angular momentum respecting the three-fold rotational symmetry. The Hamiltonian $H_{\mathrm {ring}}^{(\xi ,m)}$ exhibits a symmetry

19 $$\begin{aligned} (\Sigma \mathcal {K})^{-1} \; H_{\mathrm {ring}}^{(\xi ,m)} \, \Sigma \mathcal {K} = H_{\mathrm {ring}}^{(\xi ,m')}, \end{aligned}$$

for *m* and $m'$ satisfying $m+m' \equiv -\xi \,(\mathrm {mod}\,3)$. Here, $\Sigma$ is $\mathrm {diag}(\sigma _x\chi ,\sigma _x,\sigma _x\chi ^*,\sigma _x)$ for $\theta _1$, $\theta _3$, $\theta _4$ and $\mathrm {diag}(\sigma _x,\sigma _x\chi ^*,\sigma _x,\sigma _x\chi )$ for $\theta _2$, where $\chi$ is $e^{2i\xi \psi }\omega ^{-\xi }$, and $\mathcal {K}$ stands for complex conjugation. Thus, the states with $(m,m')=(0,-\xi )$ form twofold doublets, and belong to two-dimensional *E* irreducible representation of $D_3$ point group, while the states $m=\xi$ is non-degenerate, and belong to either of $A_1$ or $A_2$.

**Figure 6:**
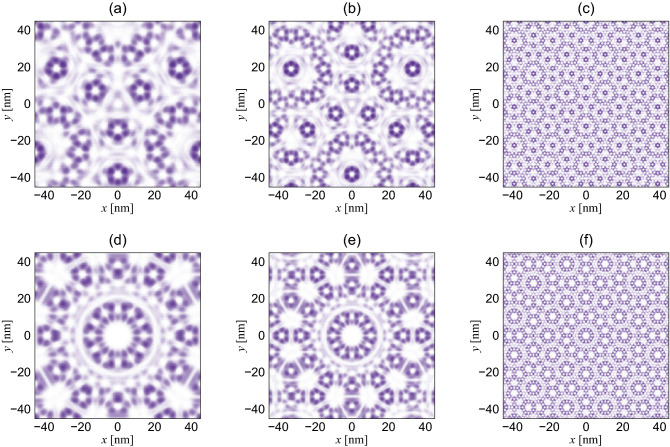
The electron probability distribution of the wave functions at $\mathbf{k}_0=\mathbf{0}$ of the double moiré superlattice (**a**)–(**c**) in the absence of the inversion symmetry, i.e., $V_{\mathrm {N}}\ne V_{\mathrm {B}}$ and $\psi \ne \pi /3 \,(\mathrm {mod}\,\pi )$, and (**d**)–(**f**) in the presence of the inversion symmetry, i.e., $V_{\mathrm {N}} = V_{\mathrm {B}}$ and $\psi \equiv \pi /3 \,(\mathrm {mod}\,\pi )$. (a) and (**d**) are for $\theta _1$, (**b**) and (**e**) are for $\theta _2$, and (**c**) and (**f**) are for $\theta _3$. We show the third state ($m=\xi$) in the valence band, since the first two states ($m=0,-\xi$) are degenerate.

Figure 6 shows the quasicrystalline wave functions of the third resonant state ($m=\xi$) in the hole side at $\mathbf{k}_0=\mathbf{0}$ on the graphene lattice. Figure 6a–c show the wave functions in a system in the absence of the inversion symmetry, i.e., $V_{\mathrm {N}}\ne V_{\mathrm {B}}$ and $\psi \ne \pi /3 \,(\mathrm {mod}\,\pi )$, such as graphene encapsulated by hBN layers, and Fig. 6d–f show those in a system with the inversion symmetry, i.e., $V_{\mathrm {N}} = V_{\mathrm {B}}$ and $\psi \equiv \pi /3 \,(\mathrm {mod}\,\pi )$, such as graphene encapsulated by a material having the same types of atoms in both sublattices. In both systems, the wave amplitude show the quasicrystalline order which is distributed on a limited number of sites in a pattern which is incompatible with the periodicity. Unlike the rotational symmetry of the double moiré pattern (12-fold), however, the wave functions in the absence of the inversion symmetry show a three-fold rotational symmetry, while those in the presence of the inversion symmetry show a six-fold rotational symmetry, which slightly resembles the probability distribution of the system with a true 12-fold rotational symmetry^22^. It should be noted that, however, the wave functions of the system with the inversion symmetry lose the six-fold rotational symmetry at $\mathbf{k}_0\ne \mathbf{0}$. Figure 6a and d, b and e, c and f show the wave functions at $\theta _1$,$\theta _2$,$\theta _3$, respectively. The length scale of the patterns is much larger than that of the quasicrystalline wave functions in quasicrystalline twisted bilayer graphene^22^, since the difference between wave vectors involved is on the order of $|\mathbf{G}_i^{\mathrm {M},(l)}|$ in the dual moiré superlattice and on the order of $|\mathbf{a}_i^*|$ in the twisted bilayer graphene ($|\mathbf{G}_i^{\mathrm {M},(l)}| \ll |\mathbf{a}_i^*|$). In addition, the systems with $\theta _1$ and $\theta _2$ show larger scale, since $|\mathbf{G}_i^{\mathrm {M},(l)}|$ is almost proportional to $\theta$ (Fig. 3c). Such an electron distribution would be prominent at the energies where the band curvature of the resonant states are large enough to give high density of states. At the energies away from the resonant states, on the contrary, we will mainly see the simple overlap of the periodic wave functions of the two typical graphene on hBN superlattice with a twist angle of $30^\circ$.

Finally, we discuss several factors that may affect the results in this work. (i) Effects of the theoretical model and parameters: In this work, we described the quasicrystalline resonant states by the effective model in the framework of a tight-binding Hamiltonian. Then, it is natural to ask how more accurate models, such as first-principle methods, affect the results. In our effective model, we describe the electronic states by using a few monolayer Bloch states which contribute significantly to the states. In first-principle methods, we get fully coupled wave functions of the states, and may analyze the contributions of each monolayer Bloch state by the projection of the wave functions to the Bloch states. The difference between the two models mainly comes from (1) the interaction between the resonant chain (Eq. 15) and the ignored monolayer Bloch states, and (2) the inaccurate parameters used in the tight-binding model (Eq. 7). Since (1) originate from the truncation of the bases, we can easily minimize the difference from (1) by using more bases in our model. However, since the energies of such truncated Bloch states are usually very different from the energy of the states which form the resonant chain, they make little change to the band dispersion. In quasicrystalline twisted bilayer graphene, for example, increasing the number of bases from the minimum 12–182 merely gives the replicas of the same bands at other wave vectors and make almost no change to the band dispersion (Fig. 6 in^22^). For (2), on the other hand, we agree that the parameters used in this work might not be accurate. The difference in the interlayer interaction strength $u_0$ will make a difference in the interaction strength within the resonant chain (Eq. 15), which affects the energy scale of the band dispersion, e.g., the energy spacing between the bands in Fig. 5a. The inaccuracy in $V_{\mathrm {B}}$ and $V_{\mathrm {N}}$ will make a difference in $\psi$ (Eq. 10), which affects the slope and the degree of anisotropy of the band dispersion. Nevertheless, the wave functions still keep the $D_3$ symmetry, and the quasicrystalline states of any similar systems, which have the same lattice symmetry and the same type of the dominant atomic orbitals, are described by the universal form Eq. (15) with slightly different parameters.

(ii) Effects of using TMDC as encapsulating materials: We can get similar quasicrystalline resonant states in the system composed of a monolayer graphene encapsulated by two TMDC layers at suitable angle. Since the metal atom in a TMDC layer is far from the interface, the dominant interlayer interaction comes from the orbital hybridization between the $p_z$ orbital of the chalcogen atom in TMDC and the $p_z$ orbital of the carbon atom in graphene. The resonant interaction in this work couples many monolayer states at various points in the Brillouin zone. Thus, to make the magnitude of every interaction which forms the resonant chain identical, so that every Bloch states can contribute with an equal footing, $t(\mathbf{q})$ (Eq. 11) should be isotropic, or has a rotational symmetry the same as $\mathbf{C}_n$. Since $t(\mathbf{q})$ between the atomic orbitals with the same magnetic quantum number is isotropic^28^, the graphene encapsulated by TMDC layers will also exhibit the resonant states respecting the quasicrystalline rotational symmetry. The difference between the lattice symmetry of the hBN layer (honeycomb lattice) and the chalcogen layer (hexagonal or triangular lattice) makes a phase difference between the *A*-to-*A* and *A*-to-*B* interaction in Eq. (9).

(iii) Effects of lattice relaxation: Incommensurately stacked atomic layers tend to exhibit local lattice relaxation, especially when the moiré pattern is sufficiently large^30,34,35^. Thus, our systems, especially the structure with an angle of $\theta _1$, will also exhibit such a relaxation. In this work, however, we did not consider such effects, since the lattice relaxation mainly influences the band opening at the main Dirac point and Brillouin zone boundaries. In more detail, the lattice relaxation changes the parameters used (e.g., the $u_0$ for *A*-to-*A* and *A*-to-*B* in Eq. (9) would become different), so there will be some differences in the details of the band dispersion [see (i) above]. However, such a difference does not change the overall symmetry and the universal equation describing the energies and wave functions of the resonant states.

(iv) Tuning conductivity by gating: It is well-known that the conventional quasicrystals are poor conductors at the energies where the quasicrystalline states are dominant (i.e., at pseudogaps). And the van der Waals quasicrystals would be also likely poor conductors at the energies where the quasicrystalline states are dominant (i.e., where there is less decoupled states), as we can expect from the large band curvatures and the wave functions distributed selectively on a limited number of sites. Thus, if we tune the Fermi energies near the quasicrystalline states by gating, which is possible only when the states are within a feasible range from the charge neutrality point as in the current system, then we will be able to tune conductivity significantly by gating.

## Conclusions

We investigated the lattice configuration and electronic structures of a double moiré superlattice of which two hexagonal *moiré patterns* are arranged in a dodecagonal quasicrystalline configuration. We first find the condition which gives a $30^\circ$ stack of the two moiré patterns in graphene encapsulated by another layers, and show that there are 0 to 4 such configurations depending on the lattice mismatch between graphene and the encapsulating layer. And we show that, although the moiré patterns satisfy a 12-fold rotational symmetry, the actual atomic lattice has only a three-fold rotational symmetry ($D_3$) if the encapsulating layers have different atomic species in the sublattices (e.g., hBN).

We then reveal the resonant interaction which brings together and hybridize twelve degenerate Bloch states of monolayer graphene as well as the band dispersion around the resonant states. Compared to the resonant states of quasicrystalline twisted bilayer graphene, of which *atomic lattices* are arranged in a dodecagonal configuration, the resonant states of double moiré superlattice lack the 12-fold rotational symmetry; they hexagonal quasicrystalline order at a specific point $\mathbf{k}_0=\mathbf{0}$ in the Brillouin zone if the encapsulating layers the same types of atoms in both sublattices, and trigonal quasicrystalline order otherwise. These unique states appear at the energies much closer to the charge neutrality point of graphene and experimentally feasible electron densities.
